# Retraction: Outcomes of Gallbladder Drainage Techniques in Acute Cholecystitis: Percutaneous Versus Endoscopic Methods

**DOI:** 10.7759/cureus.r188

**Published:** 2025-07-16

**Authors:** Rami K Morcos, Muath M Dabas, Dua F Sherwani, Javeryah R Shaikh, Abdur Rehman, Abdullah Shehryar, Roohollah Rahbani, Aima B Asghar, Yuri André Ramírez Paliza, Ramadan Khan

**Affiliations:** 1 General Surgery, Ain Shams University Hospitals, Cairo, EGY; 2 General Surgery, Ministry of Health Holdings, Dammam, SAU; 3 Surgery, The University of Jordan, Amman, JOR; 4 Internal Medicine, Jinnah Sindh Medical University, Karachi, PAK; 5 Surgery, Mayo Hospital, Lahore, PAK; 6 Internal Medicine, Allama Iqbal Medical College, Lahore, PAK; 7 Internal Medicine, Sechenovskiy Universitet, Moscow, RUS; 8 Surgery, Dr. Faisal Masood Teaching Hospital, Sargodha, PAK; 9 Plastic and Reconstructive Surgery, Universidad Peruana Cayetano Heredia, Lima, PER; 10 Internal Medicine, D.G. Khan Medical College, Dera Ghazi Khan, PAK

The Editors-in-Chief have retracted this article following an ethics complaint and internal investigation. This article was linked to an authorship-for-sale scheme, with evidence showing the article was advertised in a group offering paid authorship. Several listed authors admitted to having no role in the research or writing, confirming the use of unethical authorship practices. As a result, the credibility of the article is compromised and retraction is required.

